# Low concentrations of recombinant activated factor VII normalize coagulation patterns by reversing the changes in viscoelastic testing parameters induced by abelacimab *in vitro*

**DOI:** 10.1016/j.rpth.2025.102976

**Published:** 2025-07-22

**Authors:** Yasser Khder, Serge Côté, Klaus Peter Hoffmann, Debra Freedholm, Dan Bloomfield, Jean M. Connors

**Affiliations:** 1 Anthos Therapeutics, Cambridge, Massachusetts, USA; 2 Novartis Institutes for BioMedical Research, Basel, Switzerland; 3 Novartis Institutes for BioMedical Research, East Hanover, New Jersey, USA; 4 Hematology Division, Brigham and Women’s Hospital, Harvard Medical School, Boston, Massachusetts, USA

**Keywords:** abelacimab, anticoagulant, factor XIa inhibitor, recombinant FVIIa, ROTEM

## Abstract

**Background:**

Low doses of recombinant activated factor (F)VII (rFVIIa), used to manage bleeding in patients with severe FXI deficiency, have been proposed to bypass effects of the FXI/FXIa inhibitor abelacimab.

**Objectives:**

To test whether low concentrations of rFVIIa could abolish changes in coagulation parameters induced by abelacimab as measured by rotational thromboelastometry.

**Methods:**

Whole blood specimens obtained in citrated tubes from 6 healthy donors were incubated with 15 and 30 μg/mL of abelacimab or vehicle for 10 minutes at 37 °C. Specimens were subsequently spiked with rFVIIa at 0.5 and 1 μg/mL or vehicle. Clot formation was monitored using rotational thromboelastometry delta analyzers and the nonactivated thromboelastometry test. Clotting time (CT, in seconds), clot formation time (CFT, in seconds), α angle, etc. were measured and compared with vehicle (excipient solution containing His/His-HCl, sucrose, polysorbate 20 solution) and reference ranges provided by the manufacturer.

**Results:**

Abelacimab at 15 and 30 μg/mL concentrations increased CT by 61% and 64%, CFT by 37% and 32%, and decreased α angle by 10% and 14% compared with baseline, respectively. Adding rFVIIa at 0.5 and 1.0 μg/mL shortened CT by 21% and 38%, CFT by 33% and 49%, and increased α angle by 29% and 47%, respectively. Nonactivated thromboelastometry parameters generally remained within normal reference ranges when rFVIIa was added.

**Conclusion:**

Low concentrations of rFVIIa (0.5-1 μg/mL) corrected the effects of abelacimab as assessed by rotational thromboelastometry. Our data support using low doses of rFVIIa for bleeding management in patients treated with abelacimab.

## Essentials

• Abelacimab, a FXI/FXIa inhibitor, is associated with reduced bleeding. However, bleeding may still occur.
• This work tested whether rFVIIa can reverse the anticoagulant effects of abelacimab.
• Low concentrations of rFVIIa reversed the pharmacodynamic effects of abelacimab.
• The study supports using low doses of rFVIIa to manage bleeding in patients treated with abelacimab.

## 1 Introduction

Thromboembolic diseases are a leading cause of death and disability, accounting for 1 in 4 deaths worldwide in 2010 [1]. Warfarin and direct oral anticoagulants target coagulation factors, including those in the common pathway of coagulation, resulting in antithrombotic efficacy but with an important bleeding risk [2]. Factor (F)XI, a serine protease that originated from duplication of the prekalikrein gene, can be activated by FXII (FXIIa) through the contact activation pathway but also acts independently of contact activation when activated by thrombin via a positive feedback loop [3]. FXI is considered essential for thrombus stabilization and growth but is not essential for physiological hemostasis [[2], [3], [4]]. Subjects with severe FXI deficiency appear to be protected from thrombosis yet have a mild bleeding phenotype [5]. FXI inhibition has emerged as a promising therapeutic target for development of treatments with antithrombotic efficacy and a reduced bleeding risk.

Abelacimab is a novel, highly selective, fully human monoclonal antibody that binds tightly to the catalytic site of FXI to block generation of the activated form (FXIa). Abelacimab has demonstrated anticoagulant and antithrombotic efficacy in mice reconstituted with human FXI and in primates [6,7]. Abelacimab administered as a subcutaneous injection was superior to standard of care, low-molecular-weight heparin, in preventing venogram-confirmed venous thrombosis in a phase 2 Multicenter, RandomiZed, Active-ControLled Study to Evaluate the Safety and Tolerability of Two Blinded Doses of Abelacimab (MAA868) Compared with Open-Label Rivaroxaban in Patients with Atrial Fibrillation (AZALEA) trial in patients undergoing unilateral knee arthroplasty and was associated with a low bleeding risk [8]. In the phase 2 AZALEA randomized controlled trial, 2 doses of abelacimab (90 mg or 150 mg administered subcutaneously once monthly) in patients with atrial fibrillation and moderate to high risk of stroke were associated with substantial reduction in bleeding events compared with rivaroxaban (20 mg) [9].

While patients with congenital FXI deficiency have a mild bleeding phenotype, whether treatment with FXI inhibitors would result in a similar low risk of bleeding complications is unknown. Strategies to address bleeding that might occur in clinical trials of abelacimab were developed and based on those used to treat patients with severe congenital FXI deficiency. The recommended strategies rely on local surgical hemostatic measures and the use of antifibrinolytic agents such as tranexamic acid, especially in tissues with high fibrinolytic activity [10]. Low doses of recombinant FVIIa (rFVIIa) in combination with tranexamic acid have been used in patients with severe congenital FXI deficiency with good results, including in those undergoing major surgery [[10], [11], [12], [13]]. A similar approach was proposed as a treatment option for severe, life-threatening bleeds and in patients undergoing high bleeding risk surgery or who developed unexpected bleeding while enrolled in the AZALEA trial. Recently published data based on participants undergoing procedures in AZALEA suggest that surgery and invasive procedures can be performed safely using only local hemostatic measures and tranexamic acid, without excessive bleeding or the need for use of rFVIIa administration [14]; however, the study comprised limited data from subjects with surgery at high bleeding risk.

The aims of this study are to assess the *in vitro* effects of rFVIIa on the coagulation abnormalities induced by abelacimab in whole blood samples as measured by viscoelastic testing and to determine the concentration of rFVIIa required to adequately correct the anticoagulant effects associated with the use of abelacimab.

## 2 Materials and Methods

Human blood specimens from 6 healthy male volunteer donors were drawn by venipuncture into citrate Monovette blood sample tubes. Citrate–anticoagulated human whole blood samples were incubated with abelacimab (Novartis Mat. N° 887188; batch number 283341) or vehicle (generic clinical trials placebo for abelacimab [excipient solution containing His/His-HCl, sucrose, and polysorbate 20]) [6,15] for 10 minutes at 37 °C. Concentrations of 0.5 and 1 μg/mL of rFVIIa (eptacog alfa, Novo Nordisk, batch number ES6P812) or vehicle were then added. The following combinations of vehicle, abelacimab, and rVIIa were used: Vehicle alone (control), Vehicle + rFVIIa (0.5 μg/mL), MAA868 (15 μg/mL), MAA868 (15 μg/mL) + rFVIIa (0.5 and 1 μg/mL), MAA868 (30 μg/mL), and MAA868 (30 μg/mL) + rFVIIa (0.5 and 1 μg/mL). Final concentration of vehicle was 1% in all treated samples.

Clot formation monitoring was performed using rotational thromboelastometry testing (ROTEM delta analyzers, TEM Innovations GmbH), per manufacturer’s instructions using a nonactivated thromboelastometry (NATEM) (TEM Innovations GmbH) after recalcification of whole blood samples with the Star-tem reagent [13].

The following parameters were measured: clotting time (CT, in seconds); clot formation time (CFT, in seconds); α angle (clot formation rate, in degrees); MCF (maximum clot firmness, in millimeters); and LI30, LI45, and LI60 (lysis index; percentage): ratio of the amplitude and MCF at 30, 45, and 60 min after CT, which represents clot lysis or platelet-mediated clot retraction. Values obtained with whole blood samples were compared with reference ranges provided by the manufacturer (Reference Ranges ROTEM, Edition 2012-02-07).

Mean values and SD for NATEM parameters were calculated, and comparisons between samples spiked with abelacimab with and without rVIIa and vehicle (control group) were determined with the 2-tailed, paired Student’s *t*-test using Graph Pad Prism software.

## 3 Results and Discussion

Abelacimab concentrations of 15 and 30 μg/mL were chosen as they represent the concentrations associated with maximal pharmacodynamic response to abelacimab and are the highest projected *in vivo* therapeutic concentrations after administration of the 150-mg abelacimab dose in humans [6,15]. Both concentrations in whole blood significantly prolonged CT compared with the control group with vehicle alone by 61% and 64%, respectively, demonstrating an anticoagulant effect when compared with the manufacturer normal reference range values. Abelacimab also prolonged CFT (37% and 32% vs baseline) and reduced clot formation rate (α angle; 10% and 14% vs control).

Concentrations of rFVIIa at 0.5 and 1.0 μg/mL were used as they reflect the *in vivo* plasma concentration expected with doses of 15 to 30 μg/kg, which is much lower than the typical 90 μg/kg dose used for patients with hemophilia and inhibitors [16,17]. The addition of these low concentrations of rVIIa to abelacimab-containing samples significantly shortened the CT by 21% and 38% compared with the control group, as well as significantly decreased the CFT (33% and 49%), and decreased the clot formation time as evidenced by the increased the α angle (29% to 47%) and MCF (8% and 19%), with all 4 parameters returning to the normal reference values per the manufacturer ranges for the NATEM test. The *in vitro* effects of abelacimab and rFVIIa on whole blood NATEM assays are presented in Table and Figure.

**Table:** Nonactivated thromboelastometry results in whole blood exposed to abelacimab and activated recombinant factor VII.

NATEM-measured parameters	Manufacturer normal range	Vehicle (control group)	Vehicle	Abelacimab 15 μg/mL			Abelacimab 30 μg/mL		
rFVIIa, μg/mL		0	0.5	0	0.5	1	0	0.5	1
CT, s	300-1000	866 (83)	582 (167)^a^	1391 (528)^a^	651 (179)^a^	565 (113)^a^	1419 (477)^a^	686 (222)	567 (137)^a^
CFT, s	150-700	347 (74)	203 (53)^a^	474 (178)	233 (53)^a^	184 (40)^a^	457 (150)	220 (53)	177 (44)^a^
α angle	30-70	39 (6)	54 (8)^a^	35 (7)	50 (7)^a^	56 (6)^a^	34 (8)	52 (7)^a^	58 (7)^a^
MCF, mm	40-65	48 (4)	53 (2)	48 (2)	52 (3)^a^	57 (2)^a^	48 (5)	53 (3)^a^	56 (2)^a^
LI60, %	90%-100%	95 (4)	95 (4)	95 (3)	95 (3)	95 (3)	96 (2)	95 (5)	94 (4)

Values are shown as mean (SD).

CFT, clot formation time, CT, clotting time; LI60, lysis index at 60 minutes; MCF, maximum clot firmness; rFVIIa, recombinant activated factor VII.

a *P* < .05 vs the control group (vehicle alone).

**Figure:**
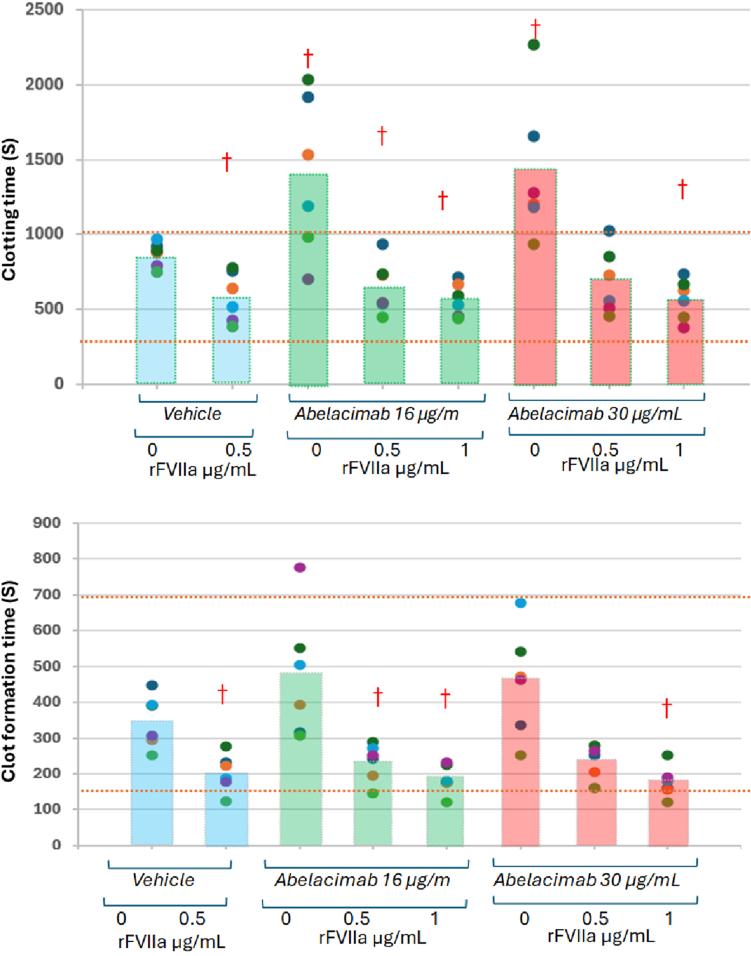
*In vitro* effects of abelacimab and recombinant activated factor VII (rFVIIa) on the nonactivated thromboelastometry parameters clotting time and clot formation time in whole blood. Bar boxes represent groups’ mean value; individual values are represented as dots. Dotted lines delimit the normal reference interval. †*P* < .05 vs the control group.

In this *in vitro* study in whole blood, abelacimab at concentrations achieved for therapeutic doses *in vivo* resulted in changes in thromboelastometry-measured coagulation parameters reflecting its anticoagulant effects compared with control samples and standard reported normal values. Low concentrations of rFVIIa abolished the anticoagulant effects of abelacimab on clotting parameters as measured by nonactivated viscoelastic testing in whole blood from healthy human donors. Concentrations of 0.5 and 1.0 μg/mL rFVIIa effectively restored normal hemostatic values, increasing the kinetics of clot formation (CFT and α angle parameters) as compared with abelacimab alone. The results did not generally exceed the manufacturer’s lower limit of the normal range, suggesting no excessive hypercoagulable effect with the concentrations of rVIIa used, either in the control vehicle sample or the abelacimab-spiked samples.

Recombinant FVIIa was approved for the treatment of bleeding episodes in patients with hemophilia A and B with inhibitors to FVIII and FIX and in those with congenital FVII deficiency [16]. The recommended dose in hemophilia A and B is 90 μg/kg every 2 hours; however, doses as low as 15 to 30 μg/kg are recommended for patients with severe FXI deficiency. Reports demonstrating efficacy for off-label uses, which may account for about 97% of rFVII use [17], include use for surgical or trauma-related bleeding; for patients with cirrhosis prior to liver biopsy or for variceal bleeding; perioperative use during orthotopic liver transplantation or liver resection; cardiac surgery; reversal of anticoagulation therapy; other coagulation factor deficiencies including von Willebrand disease, FV deficiency, and FXI deficiency; and as a rescue intervention in patients with intractable bleeding despite other therapeutic measures. High-dose off-label use of rFVIIa was found to be associated with thromboembolic complications in elderly patients with multiple comorbidites [18]. However, the use of lower doses of rFVIIa in small numbers of patients with severe congenital FXI deficiency demonstrated good efficacy with no untoward thrombotic events [[10], [11], [12], [13]].

These results provide evidence that a low concentration of 0.5 μg/mL rVIIa, corresponding to a human dose of about 15 to 30 μg/kg, can restore normal coagulation activity as measured by viscoelastic testing in whole blood with therapeutic concentrations of abelacimab while often remaining within the normal range for those coagulation parameters. These data are similar to the experience in patients with severe congenital FXI deficiency, in whom a compelling mechanistic rationale supports the use of low dose rFVIIa as a bypassing agent to achieve normal hemostasis in those with this congenital bleeding disorder requiring surgery [[10], [11], [12], [13]]. Complex high-bleed risk surgeries have been performed in patients with congenital FXI deficiency with a single low dose of rVIIa [10]. Our data provide support to the current clinical trial recommendations to use low doses of rFVIIa, eg, 15 to 30 μg/mL to bypass abelacimab effects in life-threatening bleeding and in surgery with major bleeding risk.

Our study has limitations. *In vitro* studies may not reflect observations in clinical settings; however, the lack of predictive preclinical bleeding models for abelacimab thwarts generating *in vivo* preclinical data. Our study has key differences in the type of sample studied (whole blood from individual participants) and the dose of rVIIa. Two published studies used commercially sourced platelet-poor plasma to assess *in vitro* coagulation parameters in response to FXI inhibition by the oral small molecule, milvexian, or by a commercial reagent antibody to FXI that has not been characterized and is not being used in clinical trials. In both studies, the dose of rVIIa used to reverse the effects of these FXI inhibitors was equivalent to the *in vivo* concentration of a dose of rVIIa at 90 μg/kg [19,20]. To date, in the AZALEA trial, rFVIIa has not been used in any patient who underwent invasive procedures or after trauma, despite the recommendation that rFVIIa be used if needed to control major bleeding in patients undergoing surgery with high bleeding risk or those with major trauma [14].

In conclusion, the data suggest that low concentrations of rFVIIa can restore normal hemostatic parameters without inducing excess procoagulant activity in human whole blood spiked with abelacimab. These results support the recommendation that low doses of rFVIIa of 15 to 30 μg/kg, as proposed in the ongoing clinical trials of abelacimab, can be used as treatment to bypass the anticoagulant effects in patients requiring high bleeding risk surgery and in patients with life-threating bleeds not responsive to other therapeutic measures. Further studies are required to assess the benefits and risks of recombinant FVIIa *in vivo* in those treated with the FXI/FXIa inhibitor abelacimab.
